# Ultrasound-guided thermal ablation in patients affected by Graves’ disease: a systematic review and meta-analysis

**DOI:** 10.1007/s12020-026-04740-2

**Published:** 2026-08-08

**Authors:** Elisa Gatta, Riccardo Morandi, Claudio Casella, Roberto Negro, Mario Rotondi, Carlo Cappelli

**Affiliations:** 1 Department of Clinical and Experimental Sciences, SSD Endocrinologia, University of Brescia, ASST Spedali Civili, Brescia, Italy; 2 Centro per la Diagnosi e Cura delle Neoplasie Endocrine e delle Malattie della Tiroide, University of Brescia, Brescia, Italy; 3 Department of Clinical and Experimental Sciences, Surgical Clinic, University of Brescia, ASST Spedali Civili, Brescia, Italy; 4 Department of Experimental Medicine, University of Salento, Lecce, Italy; 5 Division of Endocrinology, V. Fazzi Hospital, Lecce, Italy; 6 Department of Internal Medicine and Therapeutics, University of Pavia, Pavia, Italy; 7 Istituti Clinici Scientifici Maugeri IRCCS, Unit of Endocrinology and Metabolism, Laboratory for Endocrine Disruptors, Pavia, Italy

**Keywords:** Radiofrequency ablation, Microwaves ablation, Graves disease, Thermal ablation, Thyroid

## Abstract

**Introduction:**

Ultrasound-guided thermal ablation (U-GTA) techniques, including radiofrequency ablation (RFA) and microwave ablation (MWA), have recently been proposed as minimally invasive alternatives to surgery or radioiodine (RAI) therapy for patients with Graves’ disease (GD) who are unresponsive to antithyroid drugs. However, evidence supporting its effectiveness and safety remains limited.

**Methods:**

A PubMed/MEDLINE, Web of Science, and Scopus research updated until January 31, 2026, was performed. The review question was: What is the euthyroidism rate (outcome) one year after U-GTA (intervention) in Graves’ disease (population)? Two reviewers independently conducted the study screening, data extraction, and risk of bias assessment. A random-effects model was adopted to pool the prevalence with corresponding 95% confidence intervals.

**Results:**

Four studies comprising 82 patients (88% female) were included in the analysis. Approximately 2.4% of patients required levothyroxine replacement after U-GTA. Thyroid volume significantly decreased following treatment, and baseline thyroid volume was identified as a predictor of relapse in one study. A transient increase in anti-TSH receptor antibodies was observed one month after U-GTA, followed by a subsequent decline. No worsening or new onset of Graves’ orbitopathy was reported. Post-procedural adverse events occurred in 6.1% of patients and were transient. At 12 months, pooled euthyroidism rate was 67.0% (95% CI 54–78), with low heterogeneity (I² = 0%).

**Discussion:**

U-GTA appears to induce biochemical remission in approximately two-thirds of selected patients with persistent or relapsed GD, with a favourable short-term safety profile and preservation of thyroid function in most cases. However, remission rates remain lower than those typically reported for definitive surgical treatment, and current evidence is limited to small, non-randomized studies with relatively short follow-up. Larger prospective comparative trials with standardized protocols are required to define the role of U-GTA within current management strategies for GD.

## Introduction

Ultrasound-guided thermal ablation (U-GTA), represented by radiofrequency (RFA) and microwaves (MWA) ablation, is a highly promising technique for treating many thyroid diseases. This procedure is a minimally invasive treatment option for benign thyroid nodules, autonomously functioning nodules, and in selected cases of thyroid malignancy [1]. Both techniques induce coagulative necrosis through heat-mediated tissue destruction, although they rely on different physical mechanisms. RFA uses high-frequency alternating electrical currents (200–1,200 kHz) delivered between electrodes to generate heat through ionic friction, typically achieving temperatures between 60 and 100 °C. In contrast, MWA generates an electromagnetic field (900–2,450 MHz) that produces hyperthermia through dipole rotation of water molecules and ionic polarization, resulting in rapid tissue heating and cell death [2]. U-GTA offers several advantages over traditional treatments, including selective ablation, minimal invasiveness, quicker recovery, and repeatability [3]. Compared to thyroidectomy, U-GTA is less traumatic, more cosmetically appealing, and more cost-effective [4, 5].

Graves’ disease (GD) is the most common cause of hyperthyroidism, at least in iodine-sufficient areas. The main mechanism responsible involves autoantibodies that bind and activate the thyrotropin receptor. Its prevalence is 4 per 1000, with a five to ten-fold higher frequency in women [6]. Patients can be affected at any age, but the incidence peaks between 30 and 50 years [7–9]. Current treatments for GD include antithyroid drugs (ATD), surgery, and/or radioiodine therapy (RAI), as appropriate. Unfortunately, the remission rate for patients treated with ATD is lower than 50%, often necessitating definitive treatment [10].

Both RAI and surgery aim to achieve disease control through functional or structural removal of thyroid tissue. In this context, U-GTA has recently been proposed as a minimally invasive alternative for patients with persistent or relapsing GD. However, unlike established definitive therapies, U-GTA results in partial rather than complete tissue ablation, and its impact on long-term remission and autoimmune activity remains uncertain.

The aim of the present systematic review and meta-analysis is to evaluate the available evidence on the effectiveness of U-GTA in achieving euthyroidism at one year in patients with GD.

## Materials and methods

### Search strategy and inclusion criteria

A comprehensive literature search of the PubMed/MEDLINE, Scopus, and Web of Science databases was conducted to find significant published articles concerning the use of radiofrequency ablation in Graves’ disease. The algorithm used for the research was the following: (“radiofrequency ablation” OR “microwaves ablation” OR “thermal ablation”) AND (“graves disease”). A review question was define based on the “Population, Intervention, Comparator, Outcome” framework (PICO): What is the euthyroidism rate (outcome) one year after U-GTA (intervention) in Graves’ disease (population)?

The search was updated until January 31, 2026. Only articles in English were considered, and preclinical studies, conference proceedings, reviews, or editorials were excluded. To expand our search, the references of the retrieved articles were also screened for additional papers.

### Eligibility criteria

The eligibility criteria were chosen taking into account the review question. Studies were considered eligible if they: (1) included patients with Graves’ disease; (2) evaluated ultrasound-guided thermal ablation techniques (radiofrequency ablation or microwave ablation); (3) reported thyroid function and/or autoimmune outcomes after treatment; (4) were original human studies published in peer-reviewed journals; (5) were published in English. Exclusion criteria for the systematic review (qualitative analysis) were reviews, letters, comments, editorials, conference abstracts, animal studies, and studies without extractable outcome data. For the quantitative synthesis, only studies reporting euthyroidism outcomes at approximately 12 months after treatment were included.

### Study selection

C.C. and E.G. independently screened the titles and abstracts of all records identified through the search strategy and subsequently assessed the full texts of potentially eligible studies according to the inclusion and exclusion criteria. Any disagreement between the two reviewers was resolved through discussion and consensus.

### Reporting

**Fig. 1:**
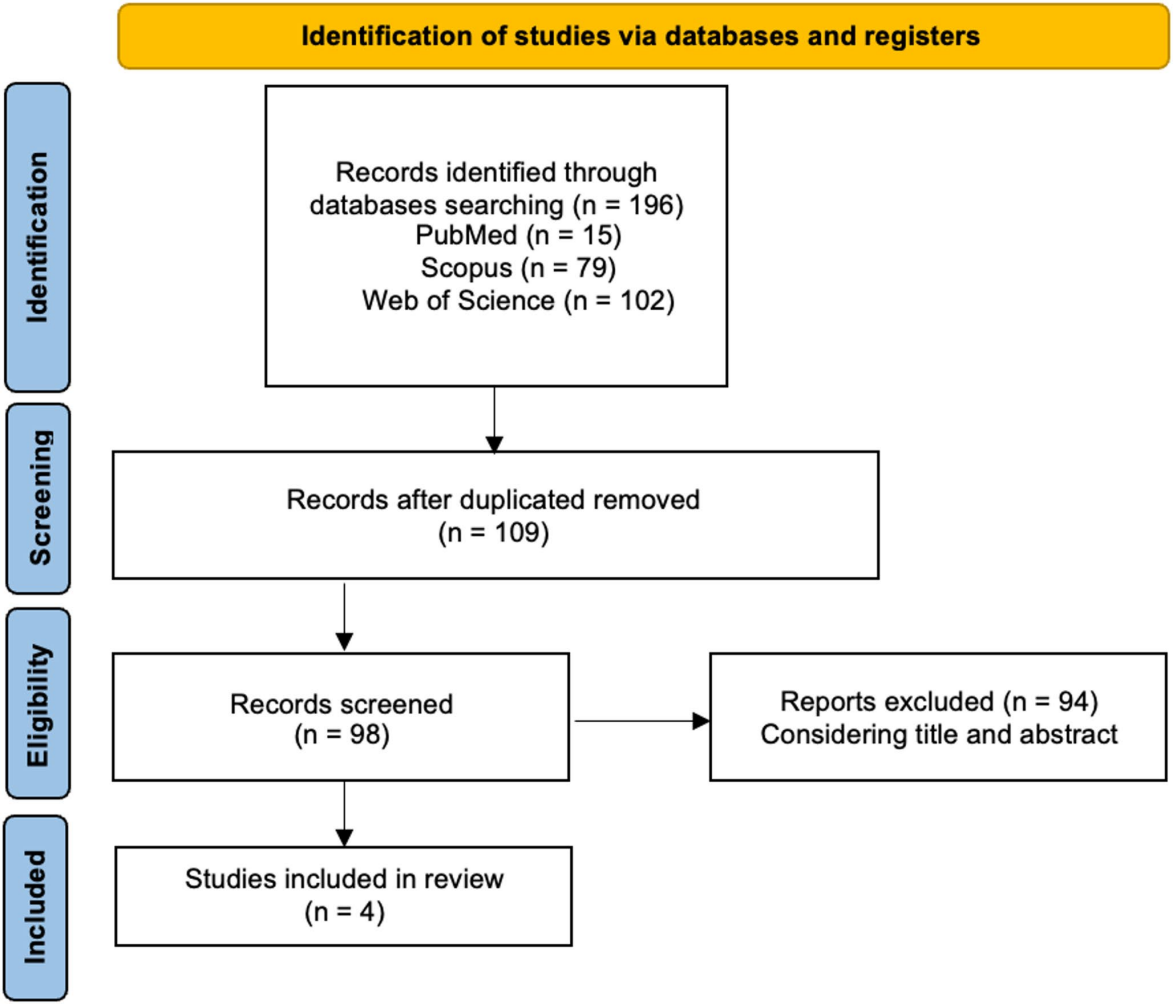
PRISMA flow diagram of study selection

The protocol of this systematic review is registered in PROSPERO (CRD420251082822) and followed the PRISMA (Preferred Reporting Items for Systematic Reviews and Meta-Analyses) guidelines [11]. The methodological quality of included prevalence studies was assessed using the Joanna Briggs Institute (JBI) Critical Appraisal Checklist for Studies Reporting Prevalence Data. The corresponding PRISMA flow diagram and JBI Report are reported in Fig. 1; Table 1.

**Table 1:** Characteristics of the human studies considered for the review

First author	Ref. *N*.	Year	Country	Study design	*N*. Pts.	Age	Sex M/F	JBI score (/10)	Overall quality
Fung	[12]	2023	China	Pilot study	15	37 (31–48)	1/14	9/10	High
Cai	[14]	2024	China	Retrospective study	29	41 (34.5–46.5)	5/24	9/10	High
Fung	[13]	2024	China	Prospective study	30	36 (29–45)	2/28	8/10	High
Zhu	[15]	2024	China	Prospective study	8	41 (27–54)	2/6	7/10	Moderate

Ref.: reference; N.: number; Pts.: patients; M: male; F: female; JBI: Joanna Briggs Institute

### Data extraction

The reviewers collected data from all the included studies, taking advantage of full-text, tables, and supplemental material concerning general study information (authors, publication year, country, study design, funding sources), patients’ characteristics (sample size, age), thermal ablation therapy (total energy applied), thyroid volume before and one year after the procedure and TSH levels one year after thermal ablation.

### Statistical analysis

The data from the included studies were utilized, considering each study’s relative importance, employing a random-effect statistical model. Furthermore, the study included the provision of 95% confidence interval values, which were subsequently visually represented through forest plots. The I-square (I^2^) index, also known as the inconsistency index, was employed to assess the level of statistical heterogeneity within the papers included in the analysis. To minimize duplication bias, potentially overlapping cohorts were carefully evaluated and only the most comprehensive or methodologically informative dataset was retained for pooled analyses. Statistical heterogeneity was considered significant if the I-square index exceeded 50%. All analyses were performed using RStudio (version 2025.05.1 + 513),

## Results

### Eligible articles

A total of 196 articles were identified through the literature search. After duplicate removal and title/abstract screening, four reports met the eligibility criteria and were included in the systematic review (Fig. 1) [12–15]. Two publications by Fung et al. reported partially overlapping cohorts [12, 13]. To avoid duplication bias, only the most comprehensive dataset was included in the quantitative synthesis. Three studies were prospective and one was retrospective, and all were conducted in China.

### Quality assessment

Overall, study quality was considered moderate-to-high. All studies used established diagnostic criteria for Graves’ disease, clearly reported patient characteristics and outcomes, and provided complete follow-up data. Nevertheless, methodological limitations included the observational design, relatively small sample sizes, single-center recruitment, and incomplete reporting of some methodological aspects, such as consecutive patient inclusion. These limitations may affect the generalizability of the findings and should be considered when interpreting the pooled estimates (Table 1).

### Qualitative synthesis

The included articles covered a total of 82 patients (88% female). The main characteristics of the studies and their results are briefly presented in Tables 1 and 2.

**Table 2:** Results and main findings of the human studies considered for the review

First author	U-GTA	Total energy applied (J)	TSH levels before U-GTA (mIU/L)	TSH levels 12 months after U-GTA (mIU/L)	TRAb levels before U-GTA (IU/L)	TRAb levels 12 months after U-GTA (IU/L)	Thyroid volume before U-GTA (mL)	Thyroid volume 12 months after U-GTA (mL)	Rate of euthyroidism 12 months after U-GTA
Cai [14]	RFA	NA	0.003 (0.003–0.003)	2.752 (0.523-4.400)	29.490 (16.960–34.910)	1.430 (0.878–5.588)	24.1 (16.9–41.2)	13.6 (10.4–22.9)	75.8%
Fung [12]	RFA	39.246 (27.530-52.426)	1.6 (0.01–3.70)	2.0 (1.25–2.25)	1.5 (1.4–3.6)	NA	16.1 (13.7–26.9)	NA	73.3%
Fung [13]	RFA	44.768 (34.238–56.504)	0.83 (0.01–2.88)	NA	3.3 (1.5–6.4)	NA	23 (15.9–34.5)	NA	60.0%
Zhu [15]	MWA	NA	NA	NA	NA	NA	40.3 (28.9–68.6)	NA	62.5%

TSH: thyroid stimulating hormone; U-GTA: ultrasound-guided thermal ablation; RFA: radiofrequency ablation; MWA: microwave ablation; TRAb: anti-TSH receptor antibodies; NA: not available

In 2023, Fung and colleagues performed a pilot study at the Hong Kong Hospital in 15 consecutive patients (93% female) with median age 37 years (IQR 31–48), with persistent or relapsed GD [12]. The total thyroid volume before RFA was 16.1 mL (IQR 13.7–26.9). Serum preablation thyroid stimulating hormone (TSH) was 1.6 mIU/L (IQR 0.01–3.70), and one year after RFA it was 2.0 mIU/L (IQR 1.25–2.25), with a disease remission rate of 79.0% at 6 months and 73.3% at 12 months. All patients had raised anti-TSH receptor antibodies (TRAb) [1.5 IU/L (IQR 1.4–3.6)]. The total energy given was 39.246 J (27.530–52.426). Among the 4 patients who relapsed after RFA, three required less ATD dose than before RFA, and no differences in clinical and demographical characteristics were found between patients who remained in remission and those who relapsed after single-session RFA. No patients suffered from treatment-related complications including vocal cord palsy, skin burn, and hematoma formation. No patients developed thyroid storm after RFA or suffered worsening of their pre-existing or new development Graves’ orbitopathy (GO).

In the subsequent year, Cai and colleagues conducted a single-centre, retrospective investigation on 29 patients (83% female) with median age 41 years (34.5–46.5), who received RFA for GD [14]. The total thyroid volume before RFA was 24.1 ml (16.9–41.2), significantly reduced [-39.7% (32.4–44.5)] one year after RFA (*p *< 0.001). A significant increase of TSH values was evidenced 12 months after RFA [0.003 ml (IQR 0.003–0.003) vs. 2.752 (IQR 0.523-4.400), respectively, *p *< 0.001] with a disease remission rate of 75.8% at 12 months. TRAb levels were significantly decrease one year after RFA [29.490 IU/L (IQR 16.960–34.910) vs. 1.430 IU/L (IQR 0.878–5.588), at baseline and 12 months after RFA, respectively, *p *< 0.001], even if a marked elevation one month following RFA [32.933 IU/L (IQR 16.973–47.780)] was evidenced (*p *< 0.001). One patient exhibited transient dysphonia and four tachycardia post-procedure.

In 2024, Fung and colleagues conducted a prospective study, including also patients of the previous pilot study [12], recruiting 30 consecutive patients (93% female) with a median age of 36 years (IQR 29–45) with persistent or relapsed GD [13]. The total thyroid volume was 23 mL (15.9–34.5), and it was the only significant factor associated with relapse after RFA (OR 1.054, *p* = .012). Serum preablation TSH levels were 0.83 mIU/L (0.01–2.88); after single-session RFA, disease remission rate was 60.0% at 12 months; among the 13 patients with relapse after RFA, 9 required a lower ATD dose and 2 received surgery without complications. Two patients were biochemically hypothyroid two years after RFA. The total energy given was 44.768 J (34.238–56.504). No treatment-related complications were observed.

In the same year, Zhu et al. reported a prospective study including eight patients with primary hyperthyroidism treated with ultrasound-guided MWA [15]. At 12 months, 62.5% of patients achieved complete discontinuation of antithyroid drugs with stable thyroid function, while the remaining patients showed a significant dose reduction. Quality of life assessment using the ThyPRO-39 demonstrated improvements across almost all domains, particularly hyperthyroid symptoms, goitre-related symptoms, and anxiety. No severe complications were observed during follow-up.

### Quantitative synthesis

**Fig. 2:**
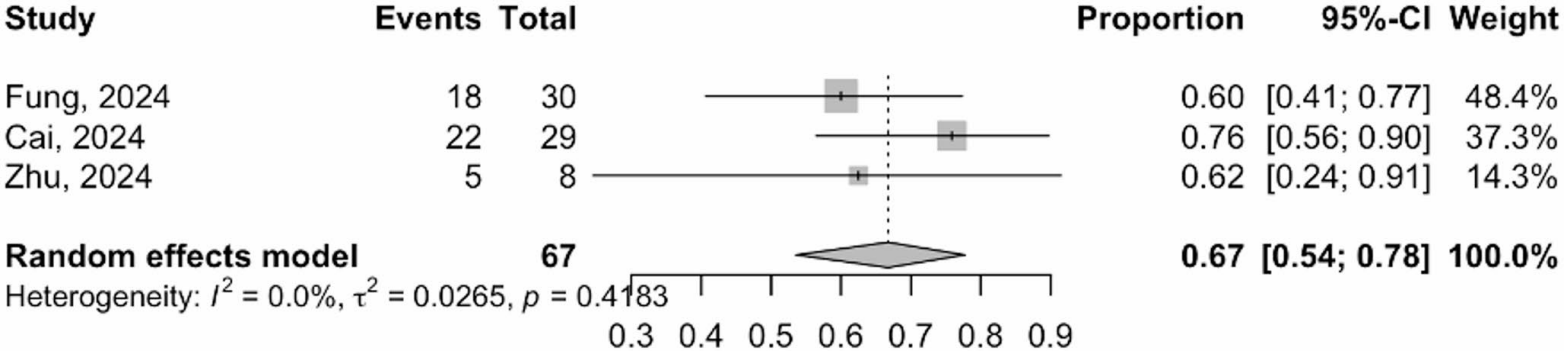
Forest plot of the meta-analysis on the rate of euthyroidism one year after ultrasound-guided thermal ablation

To evaluate the disease remission rates at 12 months after single-session U-GTA the three studies [13–15] were pooled. The meta-analysis showed that euthyroidism was reached in 67.0% of patients (IC95% 54–78) with a low heterogeneity across studies (I^2^ = 0%, *p *= 0.418) (Fig. 2).

## Discussion

Treatment of GD hyperthyroidism includes medical therapy (ATD), RAI therapy, or thyroidectomy [16]. Long-term quality of life has been reported to be comparable across treatment modalities [17]; however, important differences exist in terms of indications, contraindications, efficacy, and adverse event profiles.

Current treatment options for GD include antithyroid drugs (ATD), radioiodine (RAI), and thyroidectomy. ATD are the preferred first-line treatment in many countries, but remission rates remain below 50% and relapses are common after treatment withdrawal [16, 18]. In addition, ATD may cause adverse events ranging from mild cutaneous reactions to rare but potentially severe complications such as agranulocytosis and hepatotoxicity [18–21]. Thyroidectomy and RAI represent established definitive treatment options. Thyroidectomy provides rapid and effective disease control but is associated with surgical risks and permanent hypothyroidism requiring lifelong levothyroxine replacement [18, 22, 23]. RAI achieves disease control in most patients, although repeated treatments may be required, and hypothyroidism frequently develops during follow-up [16, 24–26]. Furthermore, RAI is contraindicated during pregnancy and breastfeeding [16, 18, 27].

Against this background, U-GTA has recently emerged as a minimally invasive approach for selected patients with persistent or relapsed GD who are unsuitable for or unwilling to undergo conventional definitive treatments.

In the present meta-analysis, euthyroidism at 12 months was achieved in 67% of patients, with low heterogeneity across studies. Only 2.4% of patients required levothyroxine replacement, suggesting partial and/or incomplete thyroid tissue ablation. In addition, thyroid size is significantly reduced within a year, resolving the aesthetic issue of large goitres without permanent scars or exposure to radiation. The safety profile reported in the included studies appears favourable, with transient post-procedural adverse events in approximately 6.1% of patients and no reported cases of permanent complications or worsening orbitopathy. Of note, although a transient increase in TRAb levels was observed one month after RFA, antibody levels subsequently declined during follow-up, suggesting a potential effect of U-GTA on the underlying autoimmune process. Since TRAb are the principal pathogenic driver of Graves’ disease, their reduction may represent an additional indicator of treatment response beyond biochemical euthyroidism. However, the available evidence remains limited and no firm conclusions regarding the immunological effects of U-GTA can currently be drawn. Moreover, compared with surgery, U-GTA has potential cosmetic and recovery-related advantages such as minor trauma, quick post-operative recovery and fewer post-operative complication [28]. At difference with RAI or ATD, U-GTA may theoretically represent an option in selected clinical scenarios where standard therapies are contraindicated [14].

However, these results must be interpreted cautiously. First, the available evidence is based on a very limited number of studies, involving fewer than 100 patients overall, predominantly from single centres and confined to a Chinese population, with short- to mid-term follow-up. Approximately 30% of patients experienced relapse or persistent disease within one year, indicating that U-GTA does not yet provide the level of definitive disease control observed with established therapies. Follow-up duration remains relatively short, and long-term durability beyond two years is largely unknown. In addition, no randomized controlled trials or direct comparisons with standard treatments are currently available.

Second, procedural standardization remains a major limitation. Unlike total thyroidectomy, where the extent of resection is clearly defined, U-GTA does not provide a predefined or universally accepted target regarding the proportion of thyroid tissue that should be ablated. None of the available studies clearly specify an a priori percentage of thyroid volume to be treated, nor do they consistently report objective intraprocedural endpoints. Key technical parameters—such as total energy delivered, energy per millilitre of thyroid tissue, proportion of gland volume ablated, or post-procedural residual viable volume—are variably reported or incompletely described. As a consequence, it is difficult to determine how much functional tissue has actually been destroyed and whether comparable biological “doses” of ablation were achieved across studies. Consequently, U-GTA is currently performed without a validated ablative threshold associated with durable remission, making it impossible to define a reproducible dose–response relationship between the extent of tissue destruction and long-term disease control. Energy delivery, treated volume, and endpoint definition therefore vary substantially, raising concerns about reproducibility, inter-operator variability, and external validity. Notably, baseline thyroid volume was identified as a predictor of relapse in one study, suggesting that incomplete ablation of functional tissue may predispose to recurrence. Currently available studies have not systematically estimated thyroid volume changes pre- and post-ablation procedure, which does not allow establishing pre-treatment thyroid volume and/or percentage of reduction thresholds predicting success or failure of the ablation procedure. Furthermore, it is likely that also TRAb serum levels may play a role in determining the final outcome.

However, given the relatively short follow-up duration (12 months), the term “relapse” should be interpreted with caution. What we observed more appropriately reflects early biochemical recurrence or persistence of hyperthyroidism rather than definitive long-term relapse. In this context, the recurrence pattern observed after U-GTA conceptually resembles the historical experience with subtotal thyroidectomy for GD, where incomplete removal of functional thyroid tissue was associated with a higher likelihood of recurrent hyperthyroidism compared with total thyroidectomy [29, 30]. Importantly, surgical data have shown that recurrences after subtotal procedures may occur even several years after treatment. Therefore, the limited follow-up in the present study may underestimate the true long-term recurrence rate after U-GTA.

The very low proportion of patients requiring levothyroxine replacement therapy (2.4%) further supports the hypothesis that a substantial amount of functional thyroid tissue remains in situ. In contrast, hypothyroidism is currently considered the expected outcome after definitive treatments for GD, particularly total thyroidectomy or radioiodine ablation. Longer follow-up studies are warranted to determine whether the initial euthyroid state observed after U-GTA remains stable over time or progressively shifts toward either recurrence or hypothyroidism.

Although U-GTA is mechanistically different from surgery, the common denominator remains the persistence of viable thyroid tissue, which from one hand represents the substrate for relapsing hyperthyroidism while on the other hand is known to delay/prevent TR Ab disappearance [31]. This analogy does not imply equivalence between the two approaches but highlights a pathophysiological principle: partial tissue ablation may intrinsically carry a risk for disease persistence. Furthermore, the transient post-procedural increase in TRAb levels likely reflects antigen release following thermal tissue destruction. While no short-term clinical consequences were reported, the long-term immunological implications of this phenomenon remain unclear.

Taken together, the results provided by the present meta-analysis indicate that U-GTA is a safe procedure that can be used also for patients with GD. However, at least at present, U-GTA should be regarded more as a surrogate rather than an alternative therapeutic strategy, being its use limited to strictly selected patients. It is likely that patients with small thyroid volumes might be more responsive to U-GTA as compared with those with large goitres.

In conclusion, U-GTA represents a potential and safe minimally invasive strategy for the management of highly selected patients with GD. Nevertheless, current evidence remains limited, remission rates appear lower than those reported for definitive surgical treatment, and—similarly to what was historically observed with subtotal thyroidectomy—lack of complete thyroid tissue ablation is likely associated with an intrinsic risk of disease recurrence. Standardized protocols and long-term comparative trials are required before U-GTA can be integrated into routine therapeutic algorithms.

## Data Availability

All relevant data is contained within the article.
